# Unwritten rules of healthcare assistance for robotic systems: multi-method workflow analysis of postoperative wound care treatments for the development of robotic assistive systems

**DOI:** 10.1007/s11548-026-03665-5

**Published:** 2026-05-19

**Authors:** Sonja Stabenow, Franziska Jurosch, Alissa Jell, Cristina Piazza, Klaus Bengler, Dirk Wilhelm

**Affiliations:** 1 Research Group MITI, School of Medicine and Health, TUM University Hospital, Technical University of Munich, Munich, Germany; 2 Department of Surgery, School of Medicine and Health, TUM University Hospital, Technical University of Munich, Munich, Germany; 3 Department of Computer Engineering, School of Computation, Information and Technology, Technical University of Munich, Munich, Germany; 4 Chair of Ergonomics, School of Engineering and Design, Technical University of Munich, Munich, Germany

**Keywords:** Material assessment, Postoperative, Wound care, FRAM, Robot, Assistance systems

## Abstract

**Purpose:**

Understanding and modeling the context of patient care interventions, such as wound dressing changes, is essential for developing intelligent robotic assistive systems. While context awareness is widely acknowledged as a requirement in robotics research, the specific information needed to create adapted behavior remains insufficiently defined for many clinical tasks. By analyzing the wound dressing procedure, which we take as a model for care provision on a surgical ward, we aim to identify and model the requirements for such systems in terms of time and used materials, as well as other contextual factors that influence the execution of the task.

**Methods:**

We collected observational and quantitative data during postoperative wound dressing procedures to capture contextual triggers and task dependencies. Material-use tracking and communication annotations provided the data foundation. Using FRAM, we developed a sociotechnical process model that represents the workflow and its requirements. Expert interviews further contextualize the boundaries for robotic assistance.

**Results:**

Human assistance is supportive in the background, and its positive impact is beyond a mere requested helper, taking for instance the mental load of preparation. Good assistance is proactive, consultative, and adapts to the care provider. For robotic systems to become embedded in the environment, they need procedural and patient-adapted behavior to interact during wound dressing.

**Conclusions:**

Intelligent robotic systems capable of supporting healthcare professionals during patient interventions have to adapt to the rules of hospital environments. Workflow models, as presented in our work, bring human expertise to robotic autonomy.

## Introduction

Worldwide, the unmet demand for qualified healthcare professionals is an acute issue for healthcare systems [1, 2]. Pressure induced by staff shortage in the medical domain has been shown in studies to lead to stress and treatment errors [3, 4]. To address this problem, clinical workflows are optimized, and robotic assistance systems promise to help alleviate the workload of staff by serving as a “helping hand”. This can relieve the mental and physical burden of healthcare professionals associated with indirect tasks [5]. Such a collaboration of reliable robotic systems and empathetic, attentive professionals for patient care may address the shortcomings of automation technology in care raised by Maibaum et al. [6]. In this medical environment, the human–robot interaction (HRI) is not only a dyadic, one-to-one interaction but also involves the person being treated [7]. This creates a multi-human interaction within the unwritten rules of care environments [8].

Like a human assistant, the robotic system requires an understanding of the context and utilizes medical domain knowledge to function properly [9]. Thereby, special attention to the workflow and interpersonal interaction is essential to work effectively [8]. While research already focuses on generating context information for surgical settings [10, 11] and descriptions of the process can be found in the literature [12], other clinical processes, which can also benefit from robotic assistance, have not yet been extensively investigated. One recurring use case is the preparation and handover of sterile materials as it is found during wound care routines [13–15]. While basic descriptions or instructions explaining the wound care process can be found [16], no comprehensive workflow analysis of this process with a focus on the assistance has yet been carried out, which is necessary for the development of intelligent robotic assistance systems.

The goal of this work is to explore how robotic assistance systems have to be designed to support patient interventions, such as wound care. By investigating the behavior patterns in care situations and structuring the treatment process into understandable treatment steps, the modeling of these interactions will derive the necessary contextual behavior and input variables for an intelligent robotic assistant of the future. Our vision is to enable robots to anticipate, based on an understanding of the temporal and contextual expectations of each task, where they need to be, when, and with what equipment.

## Related work

### Requirements for robotic systems from hospital environments

The lack of staff-centric human–robot interaction research is highlighted by the review of Babalola et al. [17], with only 32.14% of systems researching assisting healthcare staff. Nevertheless, applications with and around the patient are highly relevant, as they are the third party in this human–human–robot interaction [8]. For instance, the conclusion of the scoping review by Maalouf et al. [18] on nursing robots emphasizes that robotic systems need a clear social and legal framework to shape the robot–human relationship and provide boundary conditions. Such rules of conduct would enable safety and privacy for both agents in the interaction, including staff and patients.

While Maalouf et al. [18] call for abstract frameworks, other researchers focus on translating these needs into concrete technical specifications. On a technical perspective, Cremer et al. [19] derived hardware requirements for a robotic nursing assistive system from a analysis driven by the use cases, robotic patient sitter, and robotic patient walker. The work suggests the abilities a robotic assistive system should entail, such as speech processing, camera-based activity inference, and force requirements. Similarly, Sommer et al. [20] employed a participatory observation method to collect the transportation tasks of nurses, thereby deriving the items and identifying the need for each type of transportation task. Similar application-based collection of requirements is seen in work by Taylor et al. [5], where a prototype concept is derived from iterative co-design workshops. The authors emphasize that understanding context becomes vital, especially since the environment may be loud and voice patterns may be incomprehensible for modern audio processing.

Beyond specific hardware and task-based requirements, the success of these systems depends on their integration into existing care routines. Hornecker et al. [8] explore this through ethnographic observation, shifting the focus from isolated tasks to the technology’s impact within established care environments. The observers focused on established routines and multimodal interaction during the intervention of lifting a resident in a care home with the help of technology. One insight the authors gained was the nurse’s recurring narration of their actions to provide comfort and foster understanding for the residents. The authors argue that robotic systems in these environments are naturally socially embedded, and therefore, their ability to act or interact within the social constraints enables or hinders their functions.

The works present contributions of physical, contextual, and procedural requirements for the applications of different types of robotic systems in care environments. The collection of these requirements and derivation of robotic paradigms is essential for the development of robotic systems in healthcare environments. However, a research gap exists regarding a connected investigation of the clinical environment, manipulation techniques, and social expectations. Furthermore, the impact of procedural knowledge of patient interventions, such as the wound care process, on these requirements remains unaddressed.

### The wound care process

The aim of the wound dressing is to mechanically protect the wound, achieve the correct healing environment, and prevent the introduction of germs [16]. Changing the wound dressings is a recurrent intervention performed during the patient’s stay. As one of the most frequent healthcare-associated infections, surgical site infections significantly impede patient recovery [21]. Therefore, hospitals have implemented strategies to reduce the risk of infections and aseptically performed wound dressing changes are an important measure. Sack describes eight steps for wound care [16]:

1. Material preparation
2. Hand disinfection
3. Old wound dressing removal
4. Hand disinfection
5. Wound care (cleaning)
6. Sterile dressing application
7. Dressing fixation
8. Trash disposal

During the procedure, the direct patient-side care may be provided by various professionals (i.e., nurses or physicians), hereafter referred to as care provider (CP). The CP can opt to use either a technique that requires sterile gloves, allowing the practitioner to be in direct contact with the wound, or the so-called non-touch technique, where only sterile instruments like forceps are used to manipulate materials applied to the wound [16].

**Fig. 1:**
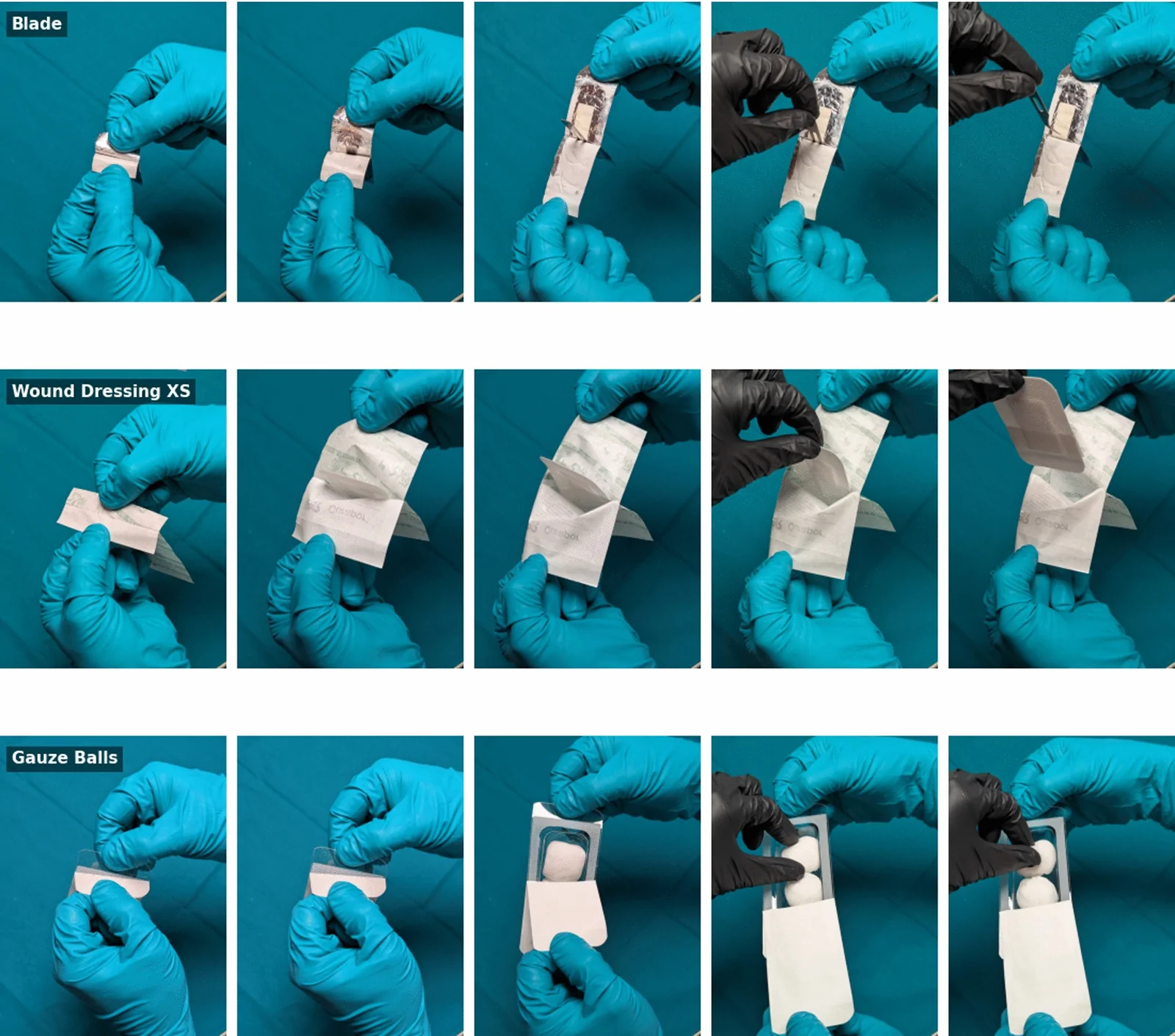
Sequence of material opening and handover for three exemplary packaged items: a blade for removing stitches, a small wound dressing, and a ball compress used for cleaning

When preparing material directly used on the wound, the sterile package must be opened, and the contents, such as swabs, must be prepared (e.g., soaked in physiological solution). Crucially, the outside of the sterile packaging must not be touched with the same gloves that are designated for direct use on the patient or the sterile field, in order to avoid contamination. Even more importantly, before accessing bulk material storage, potentially contaminated gloves must be changed and hands disinfected to prevent cross-contamination between patients [16]. Therefore, the utilization of an assistant (AST) is advantageous, especially for more complicated wound dressings, requiring multiple materials. When opening and handing over, the material is provided to the CP by the assistant as seen in Fig. 1.

## Methods

Our work uses mixed methods to establish a workflow from clinical practice and model it in relation to regulatory and hygienic guidelines. We collected material-use data during postoperative wound dressings and performed communication annotations to capture task order, contextual cues, and variations. The data collection was applied to 28 cases of wound dressing for patients with cholecystectomies and abdominal wall hernia repairs. Cholecystectomies are highly standardized with most surgeries being performed laparoscopically, while hernia repairs have a higher variability, depending on the size, localization, and genesis of the hernia. The quantitative and qualitative data from the observations of 28 cases were used to model the workflow with the functional resonance analysis method (FRAM). Finally, expert interviews were conducted to contextualize the quantitative findings in perspective with the FRAM model.

### Material tracking

**Fig. 2:**
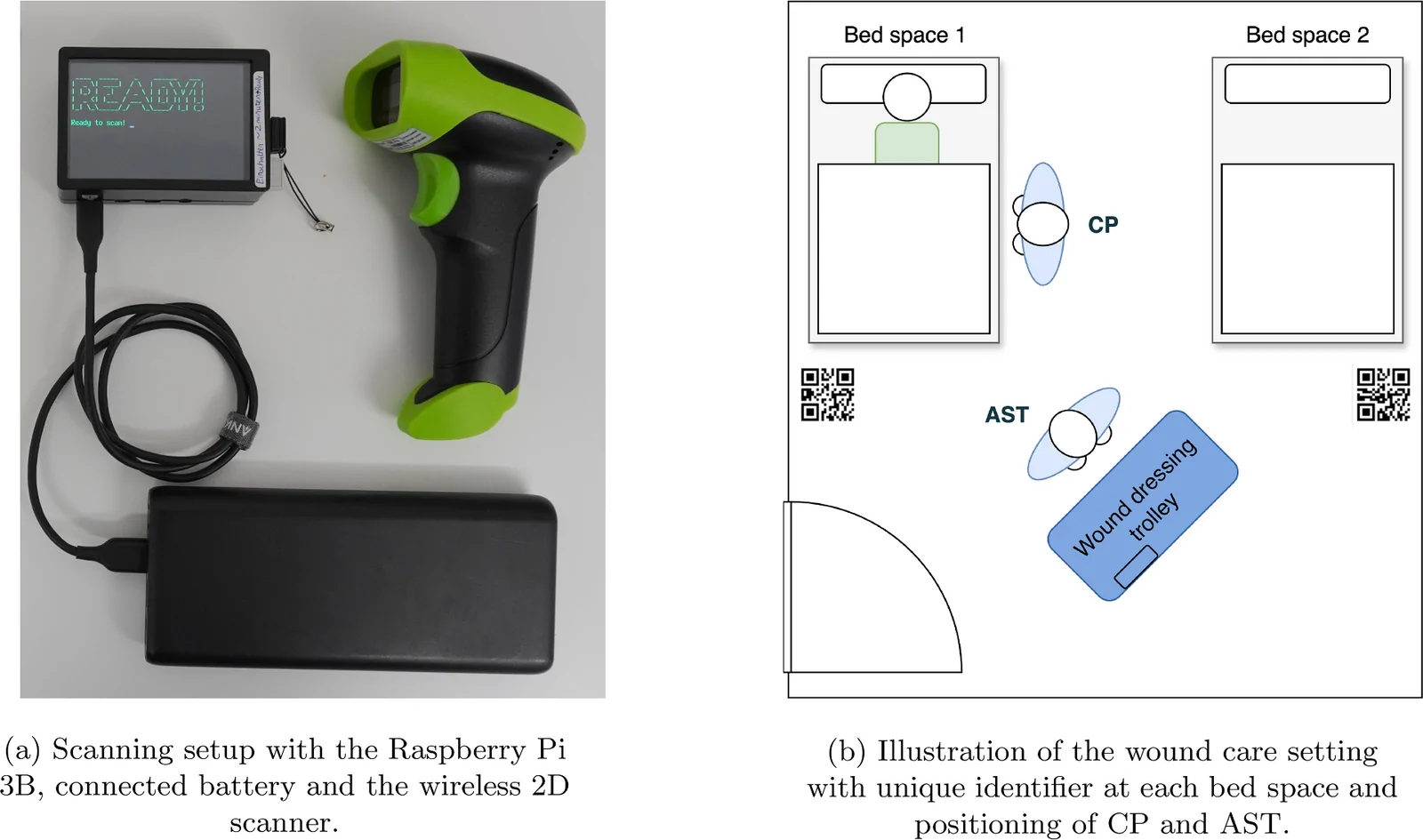
Data acquisition setup for the material tracking

Data collection was conducted at the general surgical ward of the TUM University Hospital. The study received approval from the ethics committee. Participation was voluntary, and for each consenting patient, surgical wounds were systematically documented. Furthermore, all materials used during wound care procedures were recorded. To record the frequency and timing of the used wound care materials, the material packages are scanned. For this, the dressing trolley was equipped with a scanning setup shown in Fig. 2a consisting of a wireless 2D scanner, a Raspberry Pi 3B, and a battery. Most dressing materials already have a barcode, QR code, or DataMatrix code (code) on the outer packaging that contains product information. For packaging that lacked such a code, a corresponding code was provided on the dressing trolley.

To ensure pseudonymization, a unique identifier was assigned to each bed space within the patient ward, and all collected data were systematically linked to these identifiers by scanning them at the beginning of the dressing change. An illustration of the setup is depicted in Fig. 2b.

### Communication annotation

To understand the communication during wound dressing, a communication annotation method was developed. Observations may limit attention to the most striking interactions, while subtle communication would be overlooked. Therefore, we applied an annotation to quantify communication occurrences. We used timestamped key presses recorded during the intervention, where each key represents a specific interaction between the CP and the AST. The key set was refined over four iterative cycles, with a focus on the assistance process and identifying points in time where information or material is exchanged. The initial set included keys representing the spoken time of each individual: CP, AST, and patient. A trial of this set concluded after a few observations that no information could be derived from these keys, as the conversation between the patient and the CP is nearly constant, while requests to the AST or clarification from the AST are rare and could not be distinguished in the pattern. The final key set represents a necessary trade-off between the feasibility of real-time annotation in a live situation and the maximization of derivable information, presented in Table 1.

**Table 1:** Communication key set for communication annotation

Care provider (CP)	Assistant (AST)
*Communication and interaction*	
CP request	AST asks for clarification
*Material handover*	
CP waits for AST	AST waits with material
*Task context*	
CP discards waste	AST temporarily out of room
–	AST assists at patient
*Material handling*	
CP retrieves material	AST retrieves material

### Process modeling as FRAM

Technical system development is often aligned with work-as-imagined (WAI), the formal, procedural view derived from textbooks and policies, rather than work-as-done (WAD), the adaptive, real-world clinical practice used by staff to manage complexity [22]. To accurately analyze and model the inherent variability in clinical settings, where variance in work may positively or negatively affect outcomes in terms of time, resources, and patient well-being [22], we utilize the Functional Resonance Analysis Method. FRAM is a socio-technical system modeling method that explicitly aims to develop a description of WAD by examining the work and variability as experienced in the hospital, depicting workflows as they are carried out rather than as expected [22].

As a modeling technique, FRAM has demonstrated its flexibility and effectiveness across several medical domains [23]. It is especially helpful for identifying variability, which refers to the number of possible scenarios. Knowledge about these variations is crucial for the development of an assistive system to define capabilities that support WAD [23]. The model uses six dimensions, or connection points, to describe each FRAM function in the context of the task at hand: input (I), output (O), control (C), precondition (P), time (T), and resource (R) [22]. The FRAM functions are modeled over the 28 observations and checked for compliance with literature descriptions. The resource inputs for these FRAM functions are derived from the scanned products, ensuring the process reflects the WAD.

### Expert interview

To complement the observations of the 28 dressings and findings obtained from the data analysis of WAD, semi-structured expert interviews were conducted. The sample included the following individuals: a medical student with relevant experience with wound dressing, a senior physician, and a hygiene expert. Each interviewee received an explanation of the investigation’s focus and the aim of the interview to understand the gap between WAI and WAD. The observation results were used to formulate open-ended questions. Topics included process-relevant items and assistive behavior. For each point, the interviewees were asked for their insights into the WAD and the reasons for changes in workflows. A focus was the understanding of expectations and reasons behind assistive behavior. The second half of the interview addressed the proposed robotic assistant, specifically discussing requirements and implications derived from the initial results of the analysis. This discourse allowed interviewees to provide additional input, leading to the identification of further requirements and potential implementation strategies of robotic solutions.

## Results

### Material consumption

For the development of a robotic assistant, knowing the items to be handled and opened during the process is essential, particularly when considering the special requirements how the packaging has to be opened. As exemplified in Fig. 1, the opening and handover process must adhere to sterile techniques, which necessitate the development of specialized robotic manipulation techniques. Identifying the most frequently used items enables the prioritization of developing a robotic end effector capable of opening and manipulating these objects first, as the packages vary in size, weight, geometry, and material. Furthermore, time optimization efforts can then concentrate on efficiently handling the most common items.

**Fig. 3:**
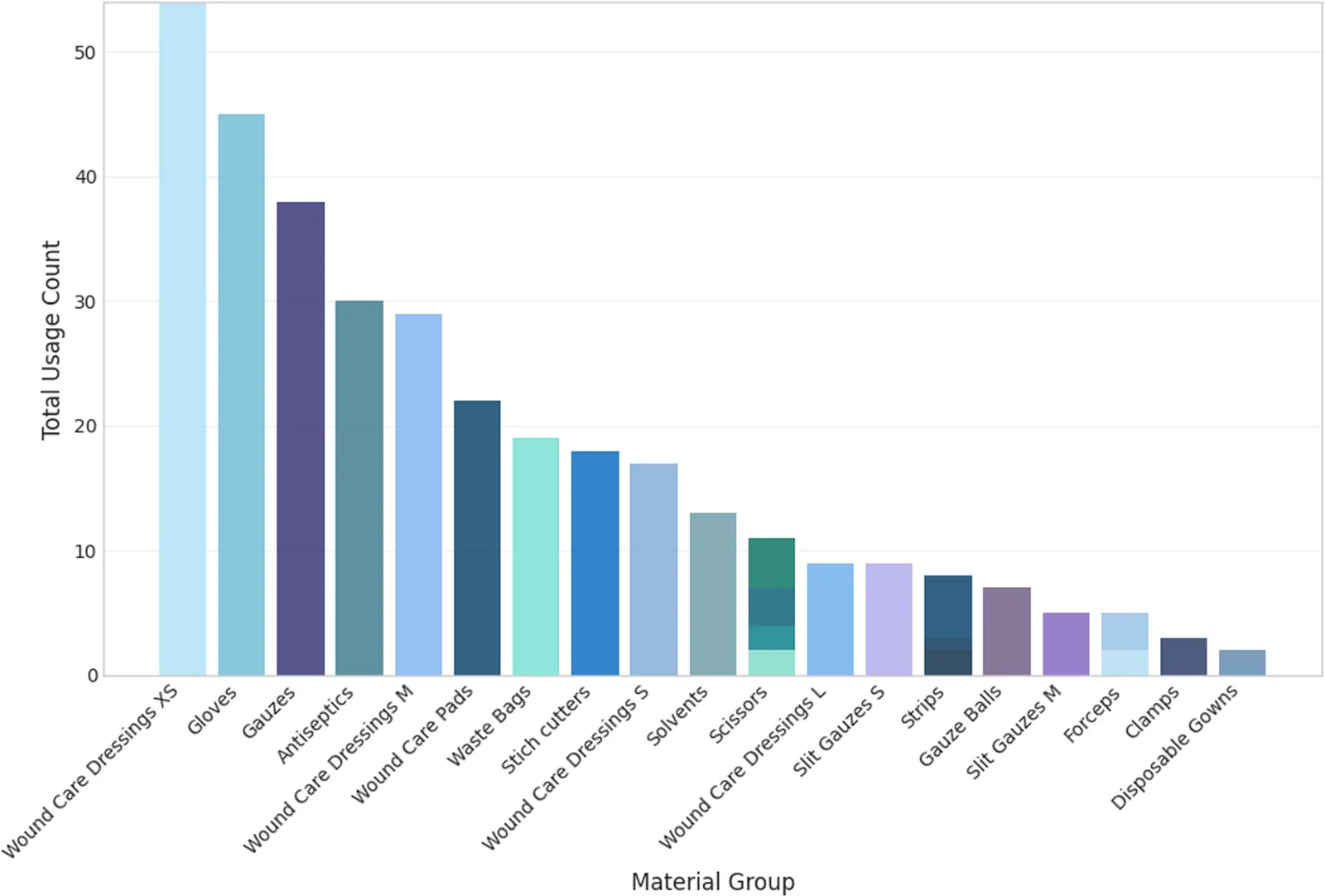
Total number of materials per material type. Scissors groups four types, indicated by different colors. Forceps include anatomical and surgical forceps, while strips group three sizes

Figure 3 presents the aggregate material usage across all recorded cases. The analysis reveals a total of 19 distinct item categories. These categories include grouped accumulation, specifically four types of scissors, two types of slit gauzes, and a combined category for anatomical and surgical forceps. The figure illustrates that small wound dressings constitute the majority of consumption, justifying their prioritization in system design, while clamps are only necessary for one type of drainage.

**Fig. 4:**
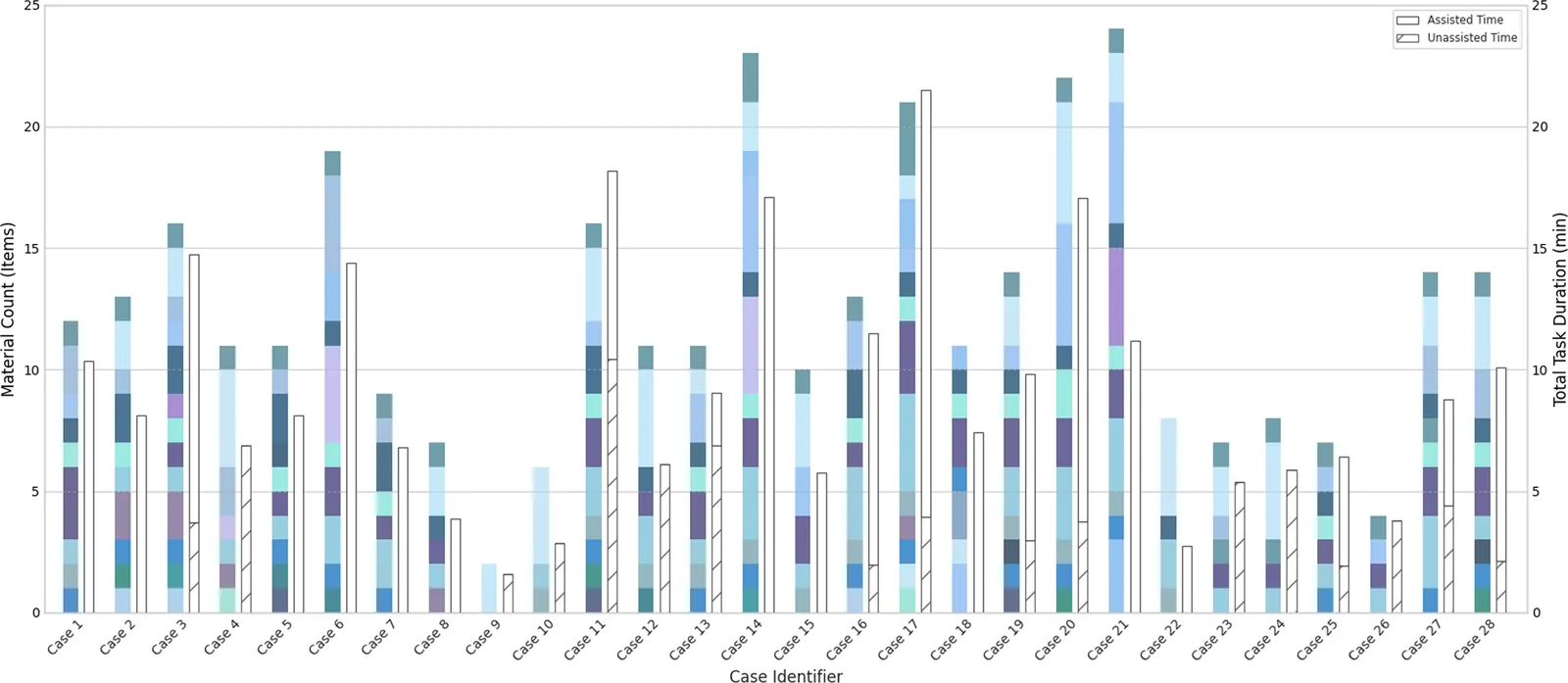
Comparative analysis of material consumption and task duration per case. The primary axis (left) quantifies the absolute material count, with stacked segments representing specific material categories color-coded for consistency with Fig. 3. The secondary axis (right) displays the total task duration in minutes, categorized into assisted and unassisted time segments

The time required for wound dressing is primarily dependent upon the extent and nature of the wounds. Figure 4 plots the duration against the prepared material count, illustrating a discernible correlation between material usage and task completion time. The same material wise color coding used in Fig. 3 facilitates the identification of a trend: Cases requiring less material variety (e.g., Cases 4, 10, and 15) exhibit shorter procedure durations.

### Workflow modeling

**Fig. 5:**
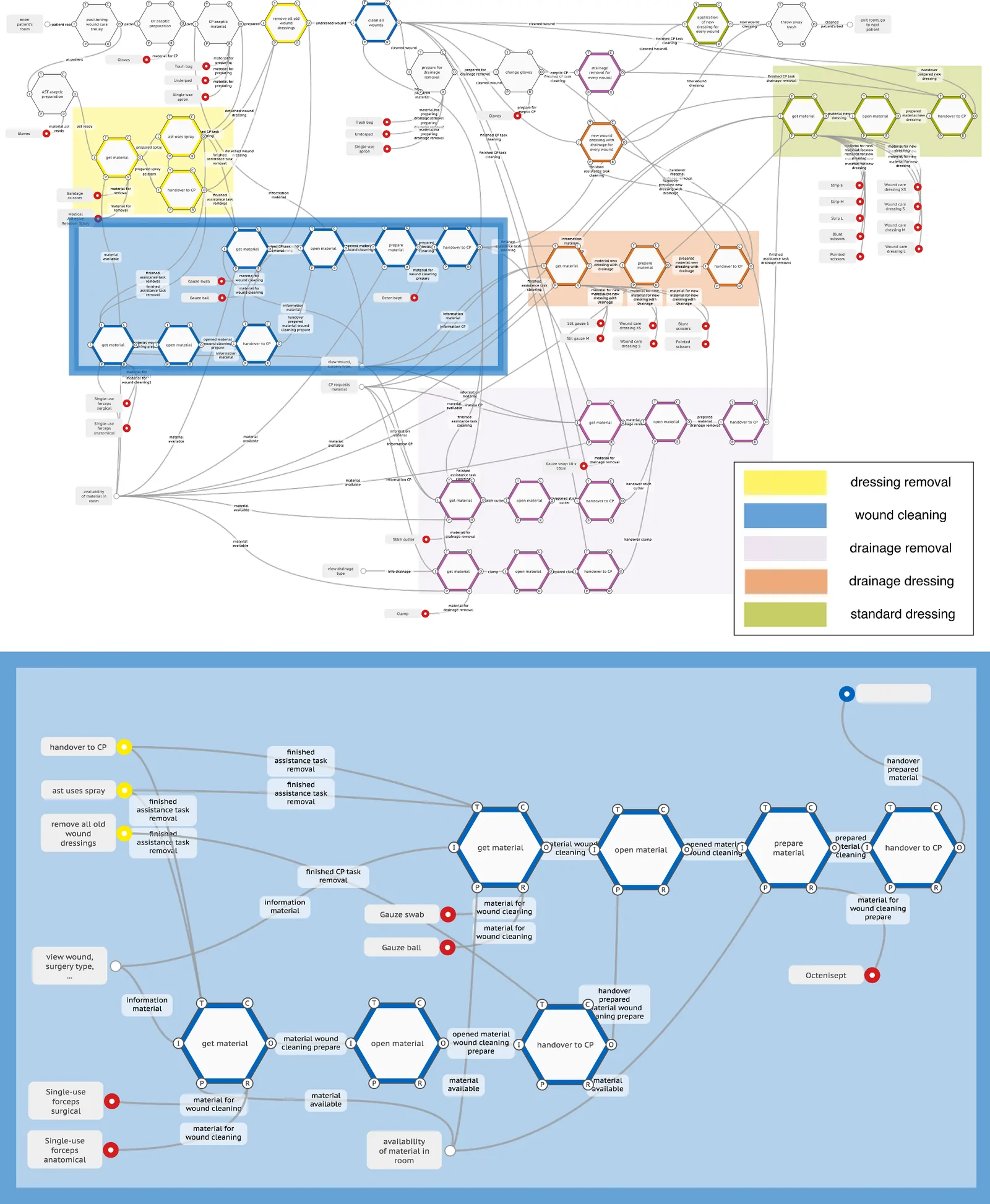
FRAM model for the assisted wound care process. The upper panel shows the system overview, while the lower panel provides the sequential FRAM functions within the preparation block, for the wound cleaning, from preparation to handover

The workflow model, developed through iterative observation and revision, is depicted in Fig. 5. We organized the primary task of the CP at the top, and the assistive actions below, color-coded by each task type. The preparatory and assistive steps performed by the AST are grouped into five main clusters: dressing removal, wound cleaning, drainage removal, drainage dressing, and standard dressing.

**Fig. 6:**
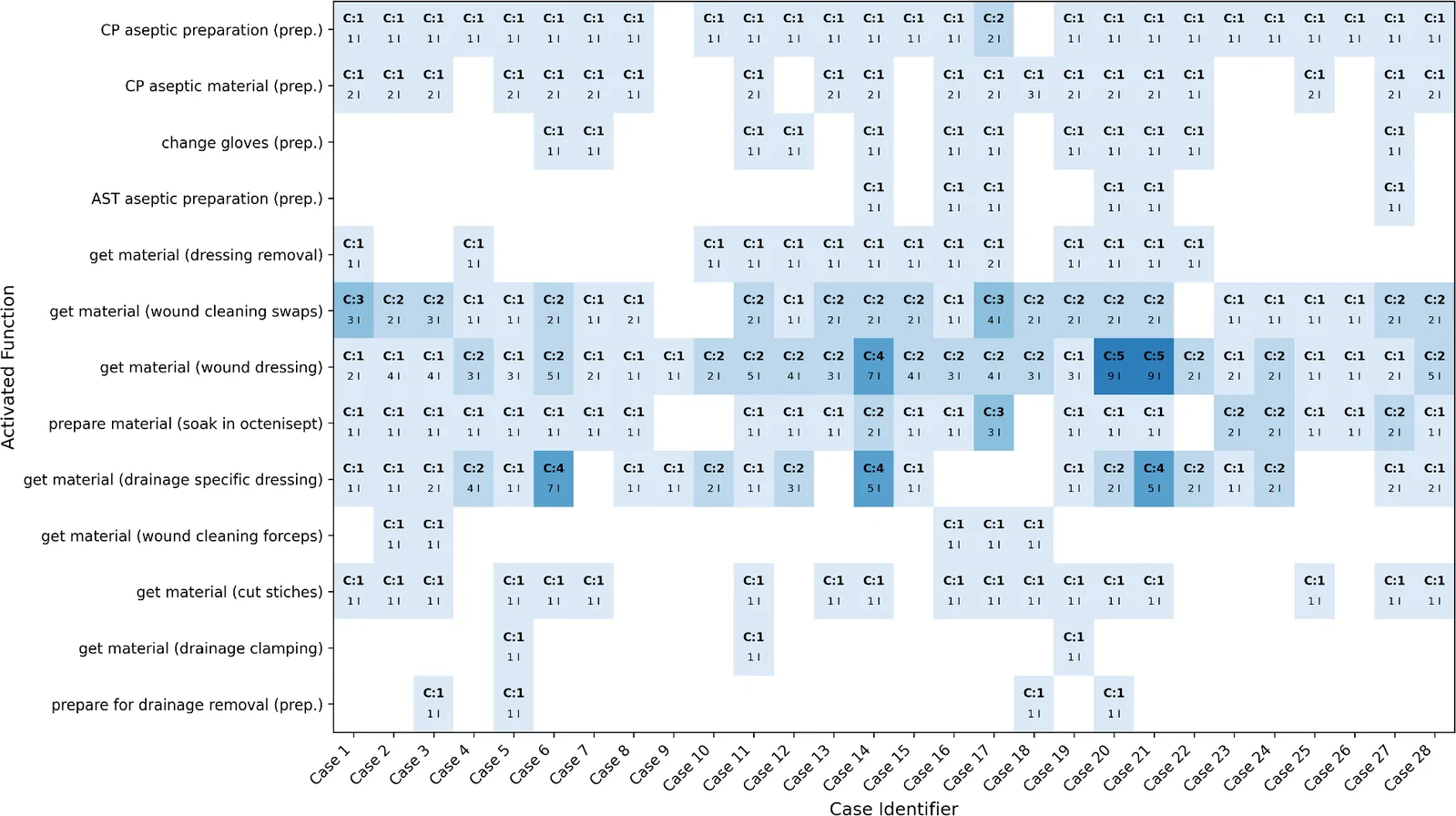
Activation of FRAM functions by case with activation counts (C) and total number of items (I) used

An excerpt illustrating one of these preparation processes is detailed in Fig. 5, which shows the fine-grained functional primitives. Materials are explicitly modeled as resource input for the respective functions, highlighted in red within the diagram. A critical aspect of the modeling is specifying the required material quantity. For each input type, it is defined whether any or all materials are required. Consequently, a sequence of functions—such as get material, open material, and handover to CP—may be activated for a bulk of items or may be activated iteratively over time based on new requests arising from the CP. A knowledgeable assistant needs fewer requests since not every material, but the sub-process itself needs to be mentioned. In Fig. 6, each activated function by material is shown. A *get material* function describes a task possible to be taken on solely by an assistant. The activation density, measured by activation counts, offers insight into how often an action needs to be repeated over the course of a single case. The analysis confirms that clinical practice follows patterns that can be modeled with a task-level activation.

Timing is a crucial factor for specifying future assistive systems, and time is tied to information. The model captures dependencies where the assistant’s action requires preconditions or time conditions. For instance, the assistant may need to wait until the wound becomes visible or until previous information leads the CP or AST to know the treatment plan and determine the necessary dressing. An assistant must be able to react to new information, prepare relevant materials, and hand them over. The more information that can be provided previously, the fewer requests become necessary.

### Communication during wound dressing

The first key set focused on the spoken communication happening during a wound dressing. We recorded the talking time of the patient, the CP, and the AST. This analysis revealed that, in most visitations, a constant exchange occurs between the patient and the CP. Information regarding the wound, its development, and the patient’s general state of well-being is discussed in addition to casual conversations about family or the weather.

**Fig. 7:**
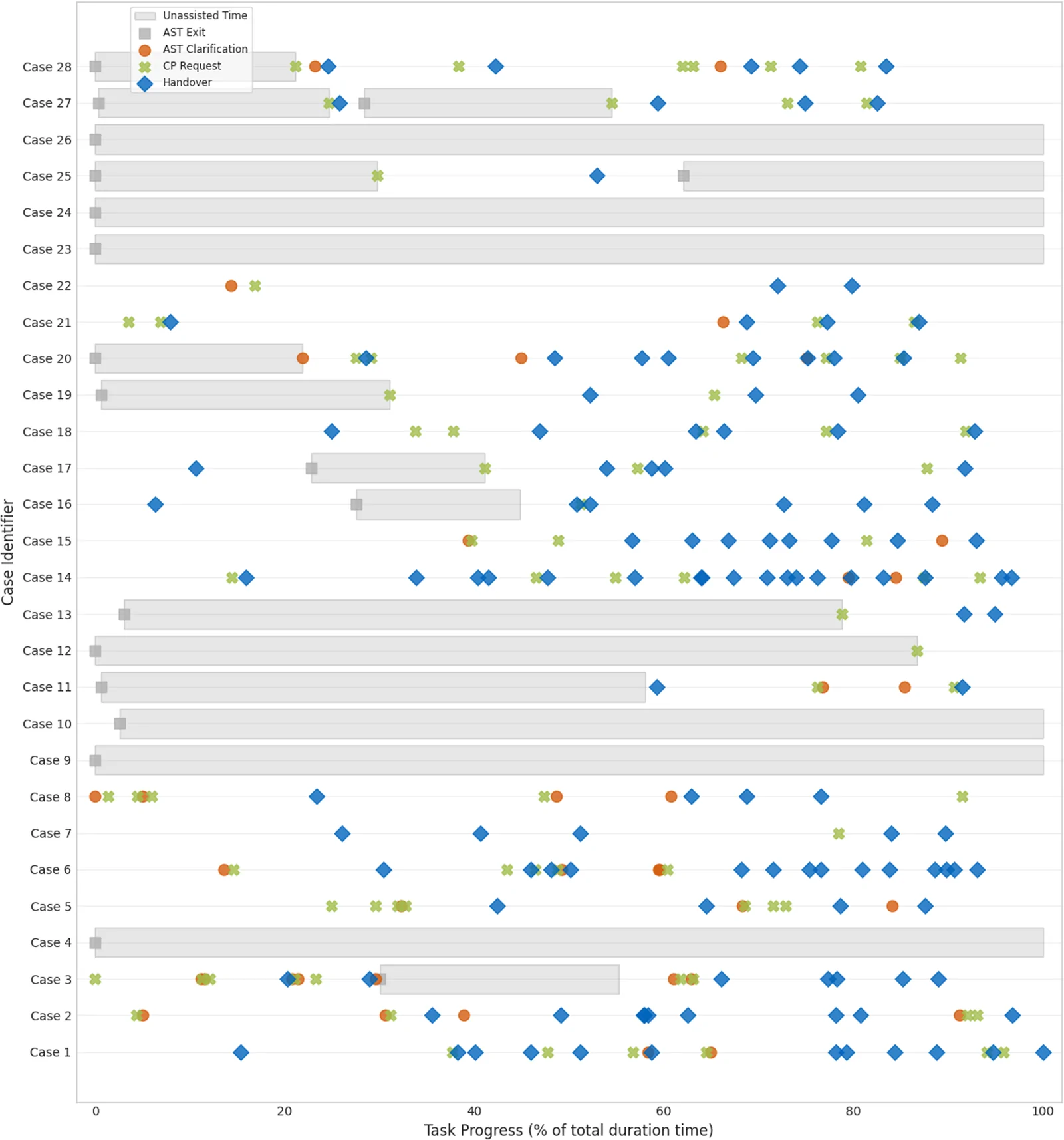
Events recorded during the wound dressing, plotted over the time span of a wound dressing, normalized to the percent of time taken

Explicit information to the AST is rare, which we focused on in our analysis. By marking the requests made to the AST and the clarifying questions asked by the AST, the direct information transfer is modeled. This analysis for the cases can be seen in Fig. 7. The data show that most communication is initiated by the CP. Expert interviews confirmed that the direction of communication from AST to CP is limited to avoid interrupting the CP during the intervention.

Furthermore, an interesting aspect to examine in the communication process is the handover of material between the AST and the CP. Our observations indicate that many handovers are decoupled from verbal communication and happen nonverbally as recorded in Fig. 7. The handover is mostly preceded by a short waiting period of the AST, standing next to the CP. Understanding the precise timing and physical position of this interaction is essential for the seamless introduction of an assistive robotic system.

### Expert perspectives on robotic assistance

Complementary to the observations and data analysis, expert interviews were conducted to address inconsistencies within the WAI as well as gain insight into the perceived role of robotic assistance. The qualitative results highlight several key insights. In general, the interviewees noted that during extensive wound dressing procedures, the presence of an assistant significantly reduces cognitive load, as it alleviates the need to anticipate all procedural contingencies during the preparation process.

The key to fluent dressing is the preparation process. To prepare and separate materials, a clean and sterile area is set up, which can be located on a tablet, a table, a cart, or the robot itself. Disinfecting between steps and when contamination occurs is a vital step, and an end effector of a robotic system should be able to do so as well.

All interviewees showed support for proactive behavior, but emphasized that the expression of proactive behavior needs to be well curated. Since patients will hear system-led corrections and might question practitioners who disregard them. Therefore, proactive outputs should be framed as suggestions to preserve the collaborative dynamic and professional credibility. A proposed interaction paradigm by the hygiene expert could be implemented. During the preparation before approaching the patient, the system goes through the necessary items like a time-out, a term used to describe a brief interprofessional pause to verify procedural requirements before a surgery begins. During the intervention, the system could approach and communicate non-verbally. For different experience levels, some steps in the process may be narrated, which could make a teaching assistant valuable in training institutions, as they will repeat protocols and help to make good clinical practice second nature.

A time-consuming task after dressing is documenting the wound status, which can be facilitated by a camera in the robotic system taking photographs of the wound. Further, storing process data of the wound change such as used material or the starting time can help documenting clinical processes.

## Discussion

We identified several behavioral principles for assistance during wound dressing.

Based on the task specification of human assistance, the robotic assistant must be able to open and provide each item while adhering to sterile technique. The robotic assistance should be optimized to provide heavily used items quickly, while outliers like drainage-specific clamps can require an additional action of the CP.

Based on the material types and consumption, an robotic assistant has to be capable of preparing several of the same items in bulk, like five XS wound dressings in a row, to keep pace with the CP.

The trained human assistant will know by experience the items that are required for the subtask (e.g. gauze swab and forceps for wound cleaning). Therefore, the robotic assistant should be capable to mirror the expertise. By proactively providing commonly grouped items, the robotic assistant should minimize iterative requests, thereby reducing the cognitive load of the CP. The FRAM-based workflow model enables these capabilities of anticipating material needs. Each function can be translated into a robot ability.

New information demands action, but some information can already be provided previously. Five main information streams are relevant for the human assistant: surgery type, previous dressing, physician order, visual cues, and CP requests. While some parameters are static, dynamic information—particularly speech—presents a significant challenge. In the patient care situation, speech is an inherently sensitive and important part of the caretaker–patient relationship. As such, it is a constant stream of information. A human assistant filters the spoken information to know what is task-specific. Therefore, assistive systems also require advanced natural language processing. Additionally, they must meet high security standards to participate in confidential conversations.

Because of the need to keep distractions to a minimum, the AST, as a listener, but background actor, should limit the number of questions or clarifications. Handovers are often signaled by the body position of the human assistant and are initiated by the movement of the CP. Therefore, robotic assistant should be able to react to non-verbal cues and express readiness as a state in a non-distractive way.

In terms of advice or directions by the assistive systems, expert interviews showed support for a proactive assistive system.

## Conclusion

The shown principles extend beyond wound dressing to any medical intervention involving an awake patient. Tied back to the proposed necessity of human–human–robot interactions by Hornecker et al. [8], we see how especially healthcare robots need to be put into a context wider than a dyadic interaction. We found key principles that a future robotic assistant should adhere to. Many observations can be directly transferred from human-to-human collaboration to robotic assistance for care providers.

Assistive robotic systems must adhere to implicit procedural rules of healthcare environments already by design. The environment, including vulnerable patients, needs to be taken into consideration.

## Data Availability

The datasets generated and analyzed during the current study are not publicly available due to restrictions related to privacy concerns for the research participants.
